# Preparation and Characterization of Yellow Peach Peel/Sodium Alginate/Glycerol Antioxidant Film Applicable for Oil Package

**DOI:** 10.3390/polym14091693

**Published:** 2022-04-21

**Authors:** Xiaomeng Lu, Zhizhou Chen, Qianyun Ma, Jianlou Mu, Xiaoyuan Li, Han Liu

**Affiliations:** 1 College of Food Science and Technology, Hebei Agricultural University, Baoding 071000, China; lxmeng1221@163.com (X.L.); maqianyun@126.com (Q.M.); jianloumu2005@sina.com (J.M.); l1305425203@163.com (X.L.); lh2436839752@163.com (H.L.); 2 College of Mechanical and Electrical Engineering, Hebei Agricultural University, Baoding 071000, China

**Keywords:** yellow peach peel, sodium alginate, RSM, antioxidant film, oil package

## Abstract

This work was dedicated to improving the utilization rate of yellow peach peel (YPP), with the addition of sodium alginate (SA) and glycerol (G) to prepare a biodegradable antioxidant film. First, the formulation of the film was optimized via response surface methodology (RSM) combined with the multi-index comprehensive evaluation method, considering physical properties including tensile strength (TS), elongation at break (E%), water solution (WS) and light transmittance (T). The RSM results displayed the best process condition was 2.50% of YPP, 0.60% SA and 0.80% of G (based on water) and compared with pure YPP film and YPP-SA film, the optimized (YPP-SA-G) film presented excellent properties with TS of 21.52 MPa, E of 24.8%, T of 21.56% on 600 nm, and WS of 41.61%, the comprehensive evaluation score of the film was 0.700. Furthermore, the films were characterized by Fourier transform infrared (FTIR) spectroscopy, scanning electron microscope (SEM), X-ray diffraction (XRD), and thermogravimetric analysis (TGA). FTIR analysis showed the main interaction of hydrogen between YPP, SA and G make the film has excellent compatibility, and the SEM images displayed that the film was dense and compacted with a little roughness. In addition, the optimized film had excellent thermal stability, suggested by TGA and XRD showed that the film’s crystal structure has been changed significantly when the SA and G were mixed in. The TPC and the ability of DPPH radical scavenging of the YPP-SA-G film was 17.68 mg·g^−1^ of GAE and 18.65%, then potential packaging applications were evaluated using soybean oil and the YPP-SA-G antioxidant film significantly decreased peroxide value (POV) to delay oil oxidation during storage. Therefore, the YPP-SA-G film is expected to provide a new theoretical basis for the use of food processing by-products and the packaging industry.

## 1. Introduction

In recent years, biobased packaging materials have obtained widespread interest due to the environmental problems caused by the use of petrochemical-based plastics. Among the biobased materials, carbohydrates, pectin, fibers, chitosan and valuable bioactive molecules, such as phenolic acids, carotenoids, flavonoids, vitamins and aromatic compounds have shown potential applications [1,2,3,4]. Yellow-flesh peaches are rich in antioxidants, dietary fiber, and trace elements, recent studies of the antioxidant composition of peaches revealed that phenolic compounds serve as major sources of potential antioxidants [5,6]. In addition, epidemiological studies have demonstrated that the consumption of yellow-fleshed peaches has many benefits for humans, such as supplementing vitamins and carotene, and also resisting free radicals in the human body to have the effect of anti-oxidation and beauty raising [7]. Yellow peaches are usually processed into cans after being peeled; however, it has been found that peels contain 2–2.5 times the concentration of total phenolic compounds as compared to flesh and whole extracts [8,9]. Usually, yellow peach peel (YPP) is directly discarded into the environment, which caused serious pollution and waste. Based on this, the utilization of agricultural by-products, such as YPP to prepare edible films seems much more profitable from the perspective of resource recycling and environmental protection [9,10,11,12].

As one of the most versatile biodegradable polymers, Sodium alginate (SA) is a linear polysaccharide derived from brown seaweed and possesses highly negative charge densities, is water-soluble, nontoxic, biodegradable and biocompatible [13,14,15,16]. However, the researchers found that alginate films generally have disadvantages, such as low mechanical strength compared to synthetic polymers [17]. In order to cover the drawbacks of single-component film, two or more components are usually expected to be combined via physical or chemical cross-linking [18,19]. Plasticizers are commonly used to improve plasticity in composite film substrates. Glycerol, due to its small molecular weight, increased the chance of chemical interactions with other substances; it is one of the most popular plasticizers used in films, in addition, it also has great stability and compatibility with hydrophilic biopolymer packaging chains [20], such as in Riku A. Talja’s study on the effects of three plasticizers (glycerol, xylitol and sorbitol) on the physical and mechanical properties of potato starch-based edible films, glycerol is more suitable for preparing film than other two alcohols [21].

Oxidation is one of the most common mechanisms of degradation in foodstuffs and limits the shelf of food [22]. Various fruit extracts from fruit peels have been tested as antioxidants in oil packaging including plum, grape seed, cranberry and pomegranate [23,24,25,26]. According to MARIÄA I. GIL’s quantitative research on the antioxidant components of different varieties of yellow peach peels, the results show that the content of polyphenols in peels is higher than that of vitamin C and carotenoids. Fruit polyphenols include a variety of antioxidants compounds, namely hydroxycinnamate, flavan-3-ols (condensed tannins), gallic acid derivatives (hydrolyzable tannins), flavonols and anthocyanins, vary widely in the phenolic composition of different fruit varieties [5]. Polyphenols have benzene rings and substituted hydroxyl groups, which can act as antioxidants, provide electrons or gas atoms, and directly participate in the removal of free radicals. The research of Lijun Sun suggested that the incorporation of apple polyphenols contributed to the antioxidant ability of the chitosan film [27].

Studies have shown that peach peel flour has a certain antioxidant capacity, which can delay lipid oxidation of cooked turkey during 12 days of refrigeration [28]. However, there are currently no studies on antioxidant films for YPP and SA. Thus, the objective of this study was to prepare antioxidant films based on YPP, SA and G, the best formula was optimized by RSM and the YPP, YPP-SA and YPP-SA-G films were characterized with respect to their mechanical, thermal, barrier properties and antioxidant capacity. Finally, the YPP-SA-G antioxidant film was used for the packaging of soybean oil to delay its oxidation to add value to different agro-industrial chains.

## 2. Materials and Methods

### 2.1. Materials

Yellow peaches were obtained from Baoding Industry Group Co., Ltd. (Baoding, Hebei, China). Sodium alginate and glycerol were all purchased from Wanke Chemical Reagent Co., Ltd. (Baoding, Hebei, China). Folinphenol, gallic acid and 2,2-diphenyl-1-picrylhydrazyl (DPPH) were purchased from Shanghai Yuanye Bio-Technology Co., Ltd. (Shanghai China). Soybean oil was purchased from a supermarket in Baoding. The main reagents were listed.

### 2.2. Films Preparation

**Figure 1:**
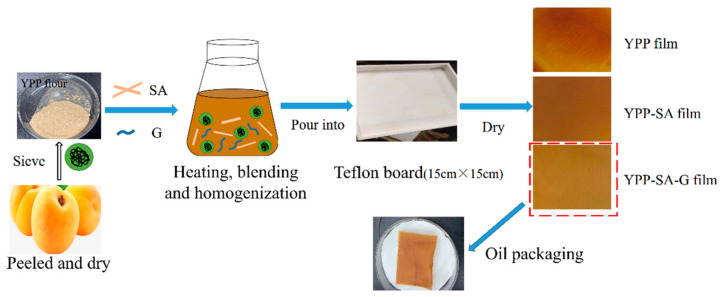
The schematic of the film-forming process.

The yellow peach peel was dried in an oven at 50 °C for 12 h, ground into flour by a high-speed mill (Shanghai, China) and then passed through a 200-mesh sieve. Figure 1 showed a schematic diagram of the film-forming process. Firstly, the YPP flour was dispersed in 100 mL of water and stirred at 60 °C for 30 min to ensure thorough mixing. Then, the film-forming solution was further processed using a high-pressure homogenizer (GJJ-0.03/100, Shanghai Noni Light Industry Machinery Co., Ltd., China) at a pressure of 40 MPa. Next, SA and G were added to the dispersion, stirred using a thermomagnetic mixer (Beijing, China) for 30 min and then degassed using a vacuum pump for 10 min. Finally, 100 mL of film-forming solution was poured into a 15 cm × 15 cm Teflon plate, the film was removed from the plate after drying at 50 °C for 5 h and incubated at 25 °C and relative humidity (56% RH) for 24 h to further evaluate the physical properties of the film.

Later, pure YPP film was prepared only with YPP flour (2.50% (W/V)), YPP-SA film was prepared using the optimized formulation without adding glycerol (YPP 2.50% and SA 0.60%), YPP-SA-G film was prepared using the optimized formulation (2.50% of YPP, 0.60% SA and 0.80% of G). These film samples were used for subsequent characterization tests.

### 2.3. Chemical Composition of Yellow Peach Peel

Protein content was measured following the GB/T 5009.5-2016 using 6.25 as a conversion factor, and the content of lipid was evaluated according to GB/T 5009.6-2016. Total dietary fiber was determined according to the methods described in GB/T 5009.10-1985. Pectin content was calculated by the method of GB/T 10742-2008. Antioxidant activity is reflected by total phenolic content and DPPH free radical scavenging rate, all determinations were performed at least in duplicate.

### 2.4. Physical Properties of the Films

#### 2.4.1. Film Thickness

A digital micrometer (Changchun, China) was used to determine the thickness of films nearest 0.001 mm. The average thickness was determined at five random positions on the films.

#### 2.4.2. Mechanical Properties

Tensile strength (TS) and elongation at break (E) values of the films were tested with an auto tensile tester (DRK101A, Derek Instrument Co., Ltd., Shandong, China) according to the method described on GB/T13022-1991. The test samples, 80 mm × 15 mm, were cut from each film and fixed on the grips of the device with a gap of 50 mm. The crosshead speed was set at 50 mm min^−1^. For each sample, at least three replicates were tested.

#### 2.4.3. Light Transmission (T)

The light transmission of the films was recorded by ultraviolet spectrophotometer (WFJ2-2000, Unocal Instrument Co., Ltd., Shanghai, China), the film was cut into 9 mm × 30 mm put directly into the cuvette to be measured. T_1_ is the transmittance of the film at 600 nm, T_0_ is the control, and the transmittance of the light without the film (its transmittance is 100%). The light transmission was calculated to Equation (1):

T = T_1_/T_0_ × 100% (1)

#### 2.4.4. Water Solubility (WS)

The WS of the films was determined according to the method of Wu [29] with slight modification. Briefly, a film (5 cm × 5 cm) was weighed (W_1_) prior to dissolution in water for 24 h at room temperature, and then removed and dried at 105 °C to a constant weight (W_2_). The WS was calculated by the following Equation (2):

WS = (W_1_ − W_2_)/W_1_ × 100% (2)

#### 2.4.5. Evaluation of the Comprehensive Performance of the Film

The films were evaluated by the Membership degree. In accordance with importance, the indexes of the films were normalized to optimize the production process. Four indices with high membership were calculated using Equation (3):

X(u) = (X_i_ − X_min_)/(X_max_ − X_min_) (3)

where X(u) is the index membership degree; X_i_ is the actual value; X_min_ is the minimum of the same index; X_max_ is the maximum of the same index.

According to the evaluated method by Hongyan [10]. The weight of TS is 40%, E is 30%, T is 10%, WS is 20%. The comprehensive score (Y) was calculated with the following Equation (4):

Y = 0.4X_1_ + 0.3X_2_ + 0.1X_3_ + 0.2X_4_ (4)

where X_1_, X_2_, X_3_, X_4_ are membership degrees of TS, E%, WS, and T%, respectively.

### 2.5. RSM Design for Optimizing the Film Properties Factors

RSM is a statistical experimental method for optimizing the preparation process, which can be used for model development, evaluation of factor effects and optimal condition factors [11]. In our study, RSM was used to study the influence factors of the content of film-forming components (including the weight of YPP, SA and G) on the comprehensive score of the film. The primary interaction and secondary effects of independent variables were optimized and evaluated using RSM. The experimental design was mainly performed at three levels (−1, 0, 1): YPP (2.0–3.0 g/100 mL), SA (0.3–0.7 g/100 mL) and G (0.6–1.0 g/100 mL) at concentrations, such as those shown in Table 1.

To analyze the experimental data, Design Expert 8.0.6 software was used to design according to the Box-behnken design (BBC), which required 17 experiments including five replicates formed at the center point. To predict the optimal point, a second-order polynomial function was established to evaluate the relationship between the yellow peach peel, sodium alginate, and glycerol concentrations and the composite score of the film.

### 2.6. Verification Test

The optimal conditions of the composite film were determined by the verification experiments, and the actual experimental data were compared with the experimental values predicted by the model to verify the validity and sufficiency of the RSM model.

### 2.7. Characterization of Films

#### 2.7.1. Scanning Electron Microscope (SEM)

The micromorphology of the films was observed by scanning electron microscope (SEM) using EDAX 4863-P (USA). The samples were covered with a thin layer of gold before observation, and all samples were photographed at 20 kV.

#### 2.7.2. Fourier Transform Infrared (FTIR)

The samples were detected and analyzed using an FTIR spectrometer (Shimadzu In-strument Co., Ltd., Beijing, China) in the attenuated total reflection mode, with a scanning range of 4000–1000 cm^−1^ and a spectral resolution of 4 cm^−1^.

#### 2.7.3. X-ray Diffraction (XRD)

The composite film was cut and placed on the X-ray diffractometer (cuka radiation) (D2Phaser, broker AXS Instrument, Co., Ltd., Beijing, China). Determination parameters: room temperature, X-ray wavelength a = 0.154 nm, Cu target, graphite monochromator, tube pressure 40 kV, Current 30 Ma, scanning range 10°~70° seventy Scanning speed 2°/min.

#### 2.7.4. Thermo Gravimetric Analysis (TGA)

TGA was carried out on a thermogravimetric analyzer (HCT-2, Hengjiu Scientific Instrument, Co. Ltd., Beijing, China) under a nitrogen gas atmosphere to evaluate the thermal stability of the specimens. Approximately 10 mg of the sample was precisely cut into small pieces and heated at a rate of 10 °C/min from room temperature to 700 °C.

#### 2.7.5. Water Contact Angle (WCA)

The hydrophilicity of all films was evaluated by contact angle measurements using a VCA dynamic contact angle tester. The contact angles of all films were measured using deionized water (5 µL).

### 2.8. Antioxidant Activity

#### 2.8.1. Total Phenolic Content (TPC)

The TPC in composite films was determined by a colorimetric reaction to the phenol method with slight modifications. Aqueous gallic acid concentrations ranging from 0 to 15 mg/mL^−1^ were used to obtain calibration curves [30]. Thin film test solutions were prepared by soaking 150 mg of thin film samples in 15 mL of distilled water for 24 h. A mix of 0.1 mL of the solution with 0.7 mL of Folinphenol reagent was added to 3 mL of 10 wt% sodium carbonate solution and then the total volume of the mixture was brought to 10 mL with distilled water, and then incubated at room temperature for 2 h in the dark. Then, the absorbance of the mixture at 765 nm was measured using an ultraviolet spectrophotometer (WFJ2-2000, Unocal Instruments Co., Ltd., Shanghai, China). The TPC in the film was expressed as mg gallic acid equivalent (GAE)/g dry weight. Each sample was tested three times and the average was taken.

#### 2.8.2. DPPH Free Radical Scavenging Activity

DPPH free radical scavenging ability is one of the important indicators to measure antioxidant activity [31]. Briefly, 1 g film samples were put into 100 mL water, stirred for 24 h and mixed until completely dissolved; 2 mL film solution was mixed with 2 mL DPPH methanol solution (0.06 mmol/L). Afterward, the absorbance of the supernatants was measured at 517 nm on an ultraviolet spectrophotometer. The DPPH free radical scavenging activity of the films was calculated as follows:

DPPH (%) = (A_0_ − A_1_)/A_0_ × 100 (5)

where A_0_ and A_1_ were the absorbance of DPPH of the control (without film sample) and film samples, respectively.

### 2.9. Application in Edible Oil

#### 2.9.1. Anti-Permeate Ability for Oil

The YPP-SA-G film was cut into bags of 50 mm × 50 mm, and 2 g of soybean oil was added to the bags to seal with starch adhesive. The bags were then placed on filter paper in a glass dish, and all oil bags were stored in a humidity-controlled room (55 ± 2% relative humidity) at 23 °C for 10 days. The weight loss rate of the oil bag is determined by weighing it against the initial weight (W_i_) at specific time intervals (W) and reported as percent weight loss.

WL (%) = (W_i_ − W)/W_i_ × 100% (6)

#### 2.9.2. Peroxide Value (POV)

Soybean oil was filled into glass test tubes, covered with YPP-SA-G film, and sealed with a string, and a sample without film was used as a control. All samples were stored at 25 °C for 30 days. According to GB/T 5009.227-2016, the peroxide value (POV) was measured every 5 days, and the triplicate results were averaged.

### 2.10. Statistical Analysis

Analysis of variance (ANOVA) was performed using IBM SPSS Statistics 26 software to assess differences between factors and levels. All data are presented as mean ± standard deviation.

## 3. Results

### 3.1. Composition of the YPP Flour

The chemical composition of YPP flour was analyzed (Table 2), it was found that the content of carbohydrates is 52.20%, of which the amount of soluble dietary fiber (pectin) is 27.30%, respectively, together with a certain amount of protein (9.11%), lipid (4.52%) and moisture (11.50%). These were the dominant components in YPP flour and compared with the research of author E. Agama-Acevedo about plantain peels [10], peels from yellow peach had a high content of dietary fiber, fat and protein than plantain peels, the high content of these biopolymers in peels could favor the subsequent film preparation towards its complete utilization [32]. Moreover, the TPC of YPP flour was 19.20 mg g^−1^ of GAE, and the DPPH free radical scavenging ability was 18.90%. This data is similar to Zhang Yueyuan’s research on the peach peels of 13 varieties [33], and the difference may be related to factors, such as peach varieties and growing periods. In addition, there are many studies on the films of other fruit peel peels, which have had a positive impact. Table 3 listed the relevant results, therefore, theoretically the YPP obtained locally has excellent film-forming properties and active action in food packaging.

### 3.2. RSM Analysis

#### 3.2.1. RSM Result

RSM was applied to investigate the linear, quadratic, and interaction effects of each independent variable (YPP, SA and G concentrations) on response values. All the response experimental data were listed in Table 3. Each value represented the mean value of three determinations.

The data in the Table 4 was met with a secondary multiple fit to obtain a regression Equation (7) with a comprehensive score (Y):

Y = 0.69 + 0.045X_1_ + 0.037X_2_ + 0.040X_3_ + 6.250 × 10 − 3X_1_X_2_ − 5.250 × 10−3X_1_X_3_ + 0.033 X_2_X3 − 0.22X_12_ − 0.051X_22_ − 0.10X_32_ (7)

The different evaluation indexes, including the F value, the lack-of-fit value, and the correlation coefficient R^2^, were presented in Table 5 to verify the model’s adequacy and accuracy. The model item *p*-value was less than 0.05, indicating that the response value was significantly related to the regression model of three factors (the concentration of YPP, SA and G). The *p*-values of the linear model (A, B, C) were less than 0.05, showing that the change of concentration in three factors of film performance were extremely significant, the order of influence was the concentration of A > B > C. The *p* of the quadratic was also significant, the influence effect was sorted as A^2^ > C^2^ > B^2^. R^2^ and adj-R^2^ were used to prove the adequacy and fit of the applied model. The correlation coefficient of the model was R^2^ = 0.9733, and the correction factor Adj-R^2^ = 0.9390, cv was 6.81%, indicating that the model can explain the comprehensive score of 93.90% of the test films, which indicated the high reliability of the models and acceptable error between the calculated and experimental results.

**Figure 2:**
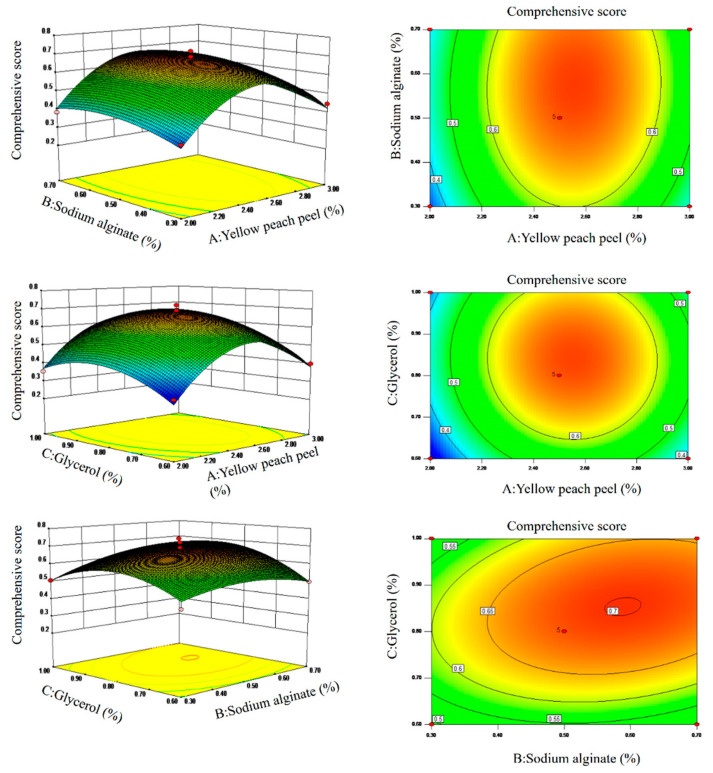
Response surface of environmental factors; Response face and Contour map between components.

Using a 3D response surface and contour map (Figure 2) we further explain the effect of interactions among independent variables (concentrations of YPP, SA and G) on the comprehensive score. The closer the bottom curve and evidently intensive contour map, which are the projection of the 3D response surface, to the ellipse, the more obvious the interaction effect and vice versa. The interaction between B and C has a combined effect by comparing the three 3D response surfaces, which is also proved by contour maps. The yellow peach peel effect was more obvious than the other two in accordance with Table 5. Figure 2 showed that in the vicinity of the near optimal process, the YPP concentration was approaching faster than others. The results further showed that the RSM model was reliable and true.

#### 3.2.2. Verification Experiments

According to the optimal variable concentrations given by the software of Design expert 8.0.6, the optimal conditions were 2.55% YPP concentration, 0.59% SA concentration and 0.85% G concentration, then the predicted value given by the software was modified to the value of experimental concentrations (2.50% YPP, 0.60% SA, 0.80% G), the verification experiment was performed to validate the reliability and validity of the model equations. The results of the verification experiments were shown in Table 6. The results between the predicted value and experimental value further demonstrated the reasonable adequacy of the response surface equations for responses and the reliability of the experiments.

### 3.3. Film Characterization

#### 3.3.1. FTIR Analysis

**Figure 3:**
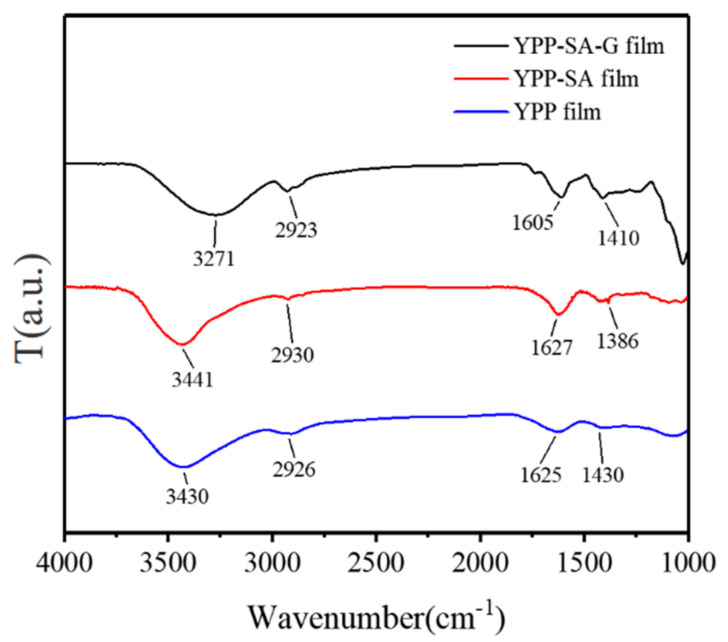
FTIR curves of various films (YPP, YPP–SA, YPP–SA–G).

The main FTIR vibrational modes of YPP, YPP-SA, and YPP-SA-G films were reported. The band frequencies between 3600–2600 cm^−1^ and 1750–1350 cm^−1^ for YPP-based films are shown in Figure 3. Due to the intermolecular and intramolecular hydrogen bonds of galacturonic acid [38], pure YPP films showed a broad, intense absorption region between 3600 and 3000 cm^−1^ associated with the -OH stretching vibration mode, and it was always associated with stretching overlap of NH bonds in amino groups [27]. Furthermore, in the range of 3000–2500 cm^−1^, the existence of moderate intensity bands is attributed to the CH, CH_2_ and CH_3_ tensile and bending vibrations. The strong absorption bands appearing at 1625 cm^−1^ are, respectively, attributed to the asymmetric stretched bands of carboxylate ions (COO^−^), while the weaker COO^−^ symmetric stretched bands can be detected at 1430 cm^−1^.

After adding SA to the YPP film, no additional peak and wavelength shift was observed, indicating that the interaction between the two compounds is more likely a physical interaction. Furthermore, for the OH group peak, after SA addition, the YPP-SA film was observed to shift to higher wavenumbers (from 3430 cm^−1^ to 3441 cm^−1^) and lower compared to the YPP film (Figure 3), which indicates that the stretching of free -OH increases due to the bonding interaction between YPP and SA. Interestingly, hydrogen bonding is the main cause of the interaction. Author Necic [39] observed similar changes in the O–H group peaks in films of pure pectin mixed with SA.

In particular, after adding G, the peak at 3441 cm^−1^ in the spectrum of the YPP-SA film was observed to shift to 3271 cm^−1^ compared to the addition of SA in the optimized YPP-SA-G film. At the same time, the peak at 1625 cm^−1^ shifts to a lower wavenumber at 1605 cm^−1^ in the YPP-SA-G film, and a strong band appears at about 2923 cm^−1^ from the CH (saturation) stretching of the glycerol chain, which indicates that the hydrogen bonding interactions between the blend components are stronger than the corresponding self-associative hydrogen bonds of pure polymers [40]. Therefore, YPP-SA-G films have better mechanical properties.

#### 3.3.2. The Physical Properties of Films

The physical properties including thickness, TS, E%, T (600 nm), WS of the YPP film, YPP-SA film and YPP-SA-G film were listed in Table 7. The thickness of the YPP-SA-G films increased insignificantly after the addition of SA and G due to the high proportion of YPP flour content in film forming solution during the drying process. In addition, the T between YPP film and YPP-SA film presented inapparent change without G content in film, but the T had a significant decline for the optimized film (YPP-SA-G), this was maybe because the good dispersion of G leads to the film not compacting in the YPP-SA substrate.

Among the mechanical properties, tensile strength (TS) and elongation at break (E) are very important properties, and different additives composing the films lead to significant differences in TS and E values. Specifically, pure YPP films had appropriate mechanical properties, with TS and E of 9.60 ± 0.24 MPa and 7.90 ± 0.35%, respectively, which were much higher than those of edible fruit and vegetable residue (FVR) films with a maximum TS of only 0.092 MPa reported in the research [9,41] This was because the pectin content of YPP flour was much higher than that of FVR flour, and thus can serve as a good film-forming substrate to prepare edible films with better mechanical properties [11]. Furthermore, the addition of SA increased the TS from 9.60 MPa to 25.21 MPa and decreased the E from 7.90% to 5.90%, and the combination of SA and pectin improved the TS due to the compatibility and chemical synergy between the components. Similar results for gelatin and SA composite films were given by Wang et al. [42] However, more G was provided in the film-forming solution, which significantly improved the flexibility of the YPP-SA film but reduced its mechanical resistance. Many studies have extensively discussed the effect of glycerol plasticizers on the mechanical properties of films [43]. Among them, Ahmadi et al. [44] reported similar results for psyllium-based edible films, that is, with increasing glycerol content, the TS value decreased and the E value increased. As shown in Table 6, after the addition of SA and G, the WS of the YPP-SA-G films decreased, which was due to the formation of new intermolecular and intramolecular bonds in the films that reduced the availability of hydroxyl groups, which in turn were limited by hydrogen bonds matrix-water interaction [13,45].

#### 3.3.3. SEM Analysis

**Figure 4:**
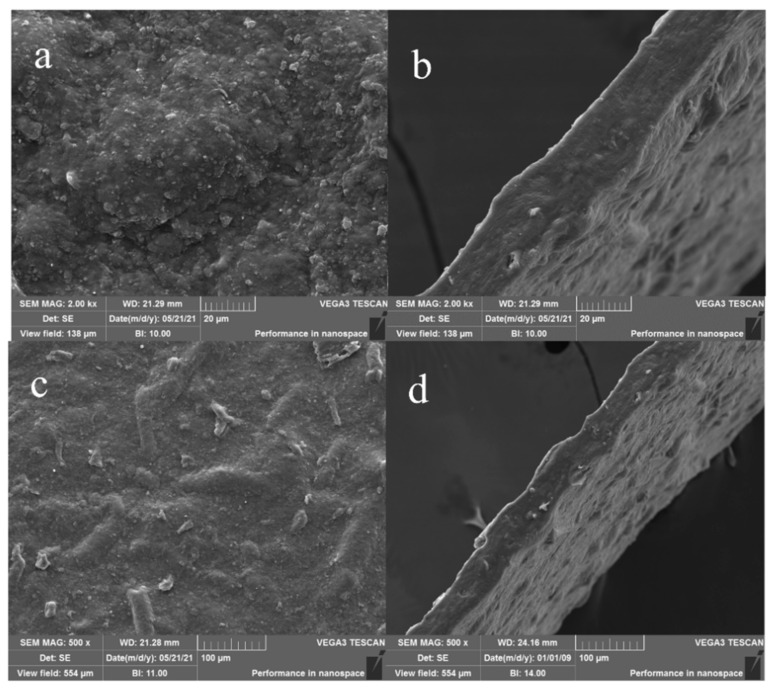
The micrographs of YPP-SA-G film. (**a**) 2000× magnification (Surface); (**b**) 2000× magnification (Cross section); (**c**) 500× magnification (Surface); (**d**) 500× magnification (Cross section).

As shown in Figure 4, SEM was applied to observe the microstructures of the surface and cross section of the optimized (YPP-SA-G) film. The smoothness and homogeneity of the surfaces presented in Figure 4a,b indicated that the components dispersed well within each other, the abundant polysaccharides, such as cellulose and pectin of YPP, which were excellent film-forming matrixes, and it had a relatively stable microstructure after film-forming under the action of SA and G [46]. Meanwhile, by using SEM at a magnification of 500×, as shown in Figure 4c,d, it could be observed that the film surface has a clear sense of particles, maybe due to the presence of insoluble celluloses in the YPP, this result was also observed by the research about pomelo peel film [29].

#### 3.3.4. XRD Analysis

**Figure 5:**
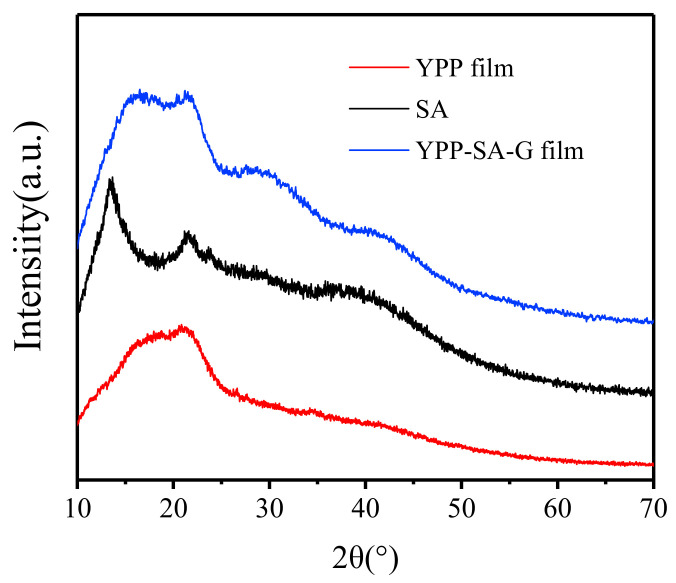
XRD patterns of various film and component.

The X-ray spectra of film samples were displayed in Figure 5. Due to the crystalline substance in the main component of YPP film, YPP-SA-G film has a higher crystallization intensity peak in the range of 16–23°. According to Gorby Gonzalles’s [45] research on yellow peach meat powder, the crystallization peak intensity indicates that it may be a slight crystallization of sugar. For another film-forming matrix, SA has an intensity peak at about 14.2° and a weak diffraction peak at 22.5° [47]. The YPP-SA-G film has a crystallization peak similar to that of YPP, which may be because yellow peach peel accounts for a larger proportion of the film-forming material, meanwhile, the diffraction peak of YPP-SA-G film at 2θ = 14.2° disappeared and shifted to 17° and the peak corresponding to 30° is gentler than that of YPP. This is the result of the interaction between the film matrixes, YPP and SA produce hydrogen bonds and destroy the crystal structure of themselves, as also proved by FTIR.

#### 3.3.5. TGA Analysis

**Figure 6:**
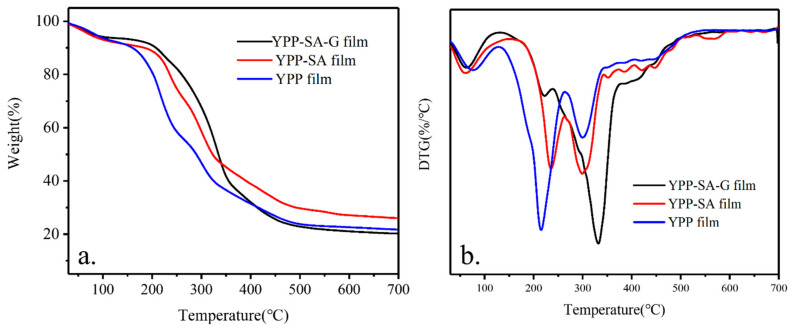
(**a**) TGA and (**b**) DTG curves of various films (YPP, YPP-SA, YPP-SA-G).

Thermogravimetric analysis is used to study the thermal properties of samples [48], the percentage of weight loss at different temperatures was analyzed (Figure 6a) It was found that the pure film degraded in three main mass loss stages within a nitrogen atmosphere. The first stage at about 30–130 °C was due to the evaporation of free and binding water in the polymer, although the weight loss was small. The main part of quality loss occurred in the temperature range of 130–450 °C which was caused by thermal degradation of biopolymers, composed mainly of pectin, cellulose, lignin, and hemicelluloses in the film [49]. The third stage (450–600 °C) showed a slow loss, possibly because of the thermal decomposition of char [50]. Similar trends were observed in YPP-SA and YPP-SA-G samples, with changes. However due to the presence of SA and G, the thermal stability of the YPP-SA and YPP-SA-G samples is higher than that of pure YPP films, respectively. The thermal stability of the YPP-SA-G film is slightly higher than that of the YPP-SA sample because the former may have more internal and internal hydrogen bonds. The temperature of maximum weight loss rate was determined as the decomposition temperature and was clearly observed in the DTG curve (Figure 6b). In all samples, the second stage allowed significant mass loss and the measurement of residues results of the samples are shown in Table 8. From these confirmations, it is demonstrated that the thermal stability of the YPP-SA-G sample is higher than that of the YPP-SA and YPP samples.

#### 3.3.6. WCA Analysis

**Figure 7:**
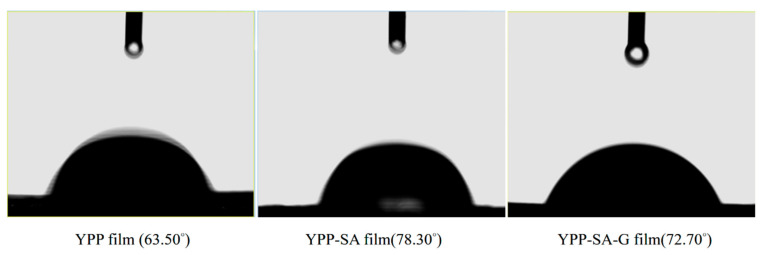
WCA of various films (YPP, YPP-SA, YPP-SA-G).

The WCA of all samples was measured to verify the change in the hydrophilicity of the mixed films after the addition of SA or SA-G, and the results were presented (Figure 7). The WCS of pure YPP film was 63.50°, which is the lowest among the samples, indicating that its surface is the most hydrophilic [51]. After adding SA or SA-G to the YPP polymer film solution, the contact angle values increased to 78.30° and 72.70°, respectively. This result indicated that the wettability of pure YPP was improved due to the hydrogen bonding force due to the utilization of more hydroxyl groups in YPP-SA and YPP-SA-G film, while the addition of G alleviates the hydrogen bonding force, which is consistent with the Changes in FTIR and TS were consistent. Therefore, the hybrid film has better hydrophobicity than pure YPP film.

### 3.4. Antioxidant Activity of the Films

The total phenolic content also associated with the antioxidant activity is shown in Table 8. The TPC of three YPP-based is not much different from that of Maria’s research on the TPC of the five varieties of yellow peach [5]. This may be the main substance responsible for the antioxidant capacity of YPP-based film. However, these data are slightly lower than the TPC in YPP flour which may be due to the influence of the temperature during the film preparation process.

DPPH radical scavenging activity is commonly used to evaluate the antioxidant effects of specific compounds or foods, the greater the DPPH radical scavenging rate, the stronger the antioxidant capacity [27]. The DPPH of the composite films were shown in Table 8. As expected, all YPP-based films showed excellent DPPH scavenging effect, and little difference was shown in the results. It could be mainly attributed to antioxidants, such as flavonoids belonging to polyphenols, carotenoids, and vitamins [29], which are in especially existed in the peels of yellow peaches. A similar result in the antioxidant activity of peach peel was also found by Zhang, who used peel flour in cooked turkey meat and proved the DPPH radical scavenging activity between 15–21 AE mg/g dry weight of the flour [28]. Therefore, it is theoretically achievable to investigate the YPP-based film in oil packaging.

### 3.5. Potential Application for Oil Packaging

#### 3.5.1. Anti-Permeate Ability for Oil

**Figure 8:**
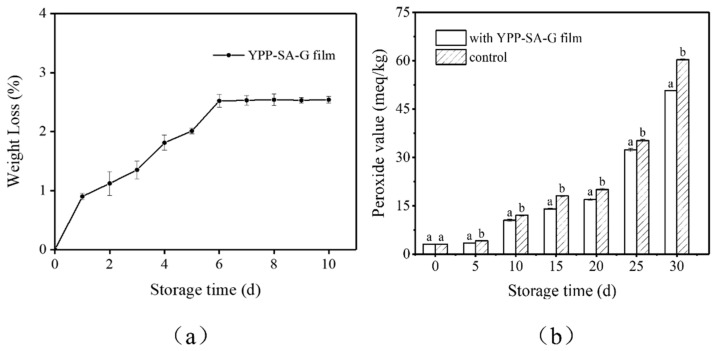
(**a**) Weight loss of oil bags made from YPP-SA-G film for 10 days storage; (**b**) The changes in peroxide value of soybean oil during 30 days storage.

To further explore the practical packaging application of YPP-SA-G antioxidant films, the anti-permeation ability of YPP-SA-G antioxidant films to soybean oil was investigated. As shown in Figure 8a, the results showed that with the prolonged storage time, equilibrium appeared on the 6th consecutive day, and at the end of the storage period (10 days), the final WL value of the oil bag was 2.54%. In addition, no oil stains were found on the filter paper under the oil bag, indicating that the membrane has good resistance to oil penetration. This can be explained by the presence of a large number of hydrophilic (or oleophobic) hydroxyl groups in the YPP-SA-G membrane, which can prevent the adsorption of oil molecules on the membrane surface. Meanwhile, the slight weight loss of the oil bag during storage may be due to the evaporation of moisture in the film.

#### 3.5.2. Peroxide Value (POV)

POV is an indicator of primary lipid oxidation. Quality characteristics of soybean oil in terms of POV were tested with control oil samples (without film) and the samples with YPP-SA-G film for 30 days for comparative study As shown in Figure 8b, the results clearly showed that POV increased throughout the storage period, while this increasing trend was slower for the YPP-SA-G film samples than for the control group. Since the soybean oil sample was directly exposed to external oxygen, the highest value for the control sample was 60.32 meq/Kg, at this time, the soybean oil with the YPP-SA-G film was found to have a lower POV (50.75 meq/Kg), because biopolymers, such as pectin and cellulose in the peel are mostly polar in nature, they are good barriers to non-polar gases, such as oxygen [49]. In addition, the results of its scavenging activity against DPPH free radicals and total phenolic content showed that the YPP-SA-G film has significant antioxidant capacity and can even delay the oxidation of soybean oil during storage. Therefore, it can be inferred that the YPP-SA-G film can be used for the packaging of oily products or liquid oily goods.

## 4. Conclusions

The YPP-SA-G antioxidant films were successfully prepared by RSM and exhibited excellent mechanical properties and the ability to retard oil oxidation. The effect of SA and G on YPP film was systematically studied. The oxidation resistance of YPP-based film did not change with the addition of SA/G, but the addition of SA greatly improved the mechanical properties of YPP film, which was opposite to the effect of G. The surface of the YPP-SA-G films were smooth and fruity, and the films had excellent hydrophilicity with the addition of SA and G. Compared with the films (YPP and YPP-SA), the YPP-SA-G film has a higher thermal degradation performance and the crystallization peak of the film changed obviously. With the increase of days, compared with the control, soybean oil wrapped in YPP-SA-G film had a lower peroxide value. Therefore, the yellow peach skin film obtained in this study has potential application value in the field of oil packaging, which is in line with the development concept of today’s green packaging and is conducive to resource recovery and environmental protection.

## Figures and Tables

**Table 1:** Experiment factor, level and coding.

Factors	Code		Coding Level	
		−1	0	1
YPP(%)	X_1_	2.0	2.5	3.0
SA (%)	X_2_	0.3	0.5	0.7
G(%)	X_3_	0.6	0.8	1.0

**Table 2:** Proximate composition of yellow peach peel (YPP) flour.

Parameters Evaluated	YPP Flour
Moisture	11.50%
Total dietary fiber	52.20%
Pectin	27.30%
Protein	9.11%
Crude fat	4.52%
Total phenolic content	19.20 GAE mg·g^−1^
DPPH	18.90%

**Table 3:** Fruits peel based edible films with their applications.

Fruit Name	Matrix	Applied on Food Items	Beneficial Effects	Ref.
Pomegranate peel flour	Mung bean protein	-	Increased total phenolic content; antioxidant activity, antibacterial capacity compared to the control film	[34]
Pomegranate peel flour	Fish Gelatin	-	Increased total phenolic content; antioxidant activity, antibacterial capacity of the film	[35]
Apple flour	CMC	Beef product	An inhibition of lipid oxidation, and efficient suppression of the growth of microbes on raw beef patties.	[36]
Orange Peel Powder	Gelatin	Cupcake	Increase in peroxide value by 3.60–4.80 (mL eq./kg fat) in refrigerated storage for 1 week and decrease in microbial growth	[37]

**Table 4:** Experiment design and responses.

Run	YPP(%)	SA (%)	G (%)	Ts/MPa	E/%	T/% (600 nm)	WS/%	Comprehensive Score
1	2.5	0.3	0.6	17.31	14.50	23.06	47.85	0.452
2	2.5	0.5	0.8	20.48	21.69	26.15	40.25	0.691
3	2.5	0.3	1.0	20.81	22.40	22.61	45.29	0.506
4	2.5	0.5	0.8	19.38	13.27	22.94	44.06	0.694
5	2.0	0.3	0.8	20.51	21.50	24.23	44.65	0.362
6	2.5	0.5	0.8	12.68	24.22	24.32	41.77	0.665
7	3.0	0.5	0.6	20.98	18.41	23.77	44.55	0.399
8	2.5	0.7	0.6	22.51	21.30	22.47	45.73	0.497
9	3.0	0.3	0.8	14.94	19.20	19.06	43.09	0.445
10	2.5	0.5	0.8	16.68	24.30	23.18	41.14	0.667
11	3.0	0.7	0.8	16.40	19.80	24.30	53.53	0.492
12	2.0	0.5	1.0	20.98	18.41	23.76	44.55	0.353
13	2.5	0.5	0.8	18.04	17.20	21.46	44.30	0.721
14	3.0	0.5	1.0	21.74	21.10	23.90	43.23	0.426
15	2.5	0.7	1.0	18.68	17.09	10.96	44.13	0.684
16	2.0	0.7	0.8	19.53	14.60	24.50	35.65	0.384
17	2.0	0.5	0.6	18.39	8.02	20.85	54.42	0.305

**Table 5:** Regression model analysis of variance table.

Sources of Variance	Quadratic Sum	Degree of Freedom	Mean Square	F Value	*p*-Value Prob > F	Significant
Model	0.31	9	0.035	28.37	0.0001	**
A-Yellow peach peel	0.016	1	0.016	13.06	0.0086	**
B-Sodium alginate	0.011	1	0.011	8.69	0.0215	*
C-Glycerol	0.012	1	0.012	10.18	0.0153	*
AB	0.000156	1	1.526 × 10^−4^	0.13	0.7316	
AC	0.00011	1	1.103 × 10^−4^	0.090	0.7730	
BC	0.004422	1	4.422 × 10^−3^	3.61	0.0994	
A^2^	0.20	1	0.2	159.34	<0.0001	**
B^2^	0.011	1	0.011	9.08	0.0196	*
C^2^	0.043	1	0.043	35.32	0.0006	**

“**”represented extremely significant (*p* < 0.01); “*”represented signifificant (*p* < 0.05).

**Table 6:** Predicted and experimental data for the responses at the optimum point.

Index	Thickness/(mm)	TS/(MPa)	E(%)	T(%) 600 nm	WS (%)	Comprehensive Score
Predict value	0.067 ± 0.007	21.03 ± 0.65	25.90 ± 0.12	22.46 ± 0.50	41.93 ± 0.61	0.701
Experiment value	0.066 ± 0.005	21.52 ± 1.10	24.80 ± 0.50	21.56 ± 0.92	41.61 ± 0.74	0.700

**Table 7:** The physical properties of the YPP, YPP-SA, YPP-SA-G films.

Index	Thickness (mm)	TS (MPa)	E%	T (600 nm)	WS (%)
YPP-SA-G film	0.066 ± 0.005 a	21.52 ± 1.10 b	24.8 ± 0.50 a	21.56 ± 0.92 b	41.61 ± 0.74 c
YPP-SA film	0.063 ± 0.002 b	25.21 ± 0.89 a	5.90 ± 0.50 c	25.65 ± 0.63 a	45.73 ± 0.82 b
YPP film	0.062 ± 0.003 b	9.60 ± 0.24 c	7.90 ± 0.35 b	25.52 ± 0.65 a	48.04 ± 1.41 a

Different superscript letters (a, b, c) in the same column indicate significant differences (*p* < 0.05).

**Table 8:** Antioxidant capacity and thermal properties of all prepared samples.

Index	TPC (GAE mg·g^−1^)	DPPH (%)	Residue (%)	^1^ DTG (°C)	^2^ DTG (°C)
YPP-SA-G film	17.68 ± 0.01 ^b^	18.65 ± 0.11 ^a^	20.20	223	332
YPP-SA film	17.80 ± 0.02 ^a^	18.45 ± 0.21 ^ab^	26.03	234	299
YPP film	17.82 ± 0.01 ^a^	18.35 ± 0.01 ^b^	21.71	214	301

Different superscript letters (a, b) in the same column indicate significant differences (*p* < 0.05): “^1^” “^2^” represented the temperature corresponding to the maximum decomposition rate of the two stages.

## Data Availability

Not applicable.
